# Role of forward and reverse signaling in Eph receptor and ephrin mediated cell segregation

**DOI:** 10.1016/j.yexcr.2019.04.040

**Published:** 2019-08-01

**Authors:** Zhonglin Wu, Tim G. Ashlin, Qiling Xu, David G. Wilkinson

**Affiliations:** The Francis Crick Institute, London, NW1 1AT, UK

**Keywords:** Eph receptor, Ephrin, Cell signaling, Contact repulsion, Cell segregation, Border sharpening

## Abstract

Eph receptor and ephrin signaling has a major role in segregating distinct cell populations to form sharp borders. Expression of interacting Ephs and ephrins typically occurs in complementary regions, such that polarised activation of both components occurs at the interface. Forward signaling through Eph receptors can drive cell segregation, but it is unclear whether reverse signaling through ephrins can also contribute. We have tested the role of reverse signaling, and of polarised versus non-polarised activation, in assays in which contact repulsion drives cell segregation and border sharpening. We find that polarised forward signaling drives stronger segregation than polarised reverse signaling. Nevertheless, reverse signaling contributes since bidirectional Eph and ephrin activation drives stronger segregation than unidirectional forward signaling alone. In contrast, non-polarised Eph activation drives little segregation. We propose that although polarised forward signaling is the principal driver of segregation, reverse signaling enables bidirectional repulsion which prevents mingling of each population into the other.

## Highlights

• Polarised forward and reverse signaling by EphB2 and ephrinB1 drives strong cell segregation and border sharpening.
• Disruption of reverse signaling in ephrinB1-6F mutant leads to decrease in cell segregation.
• Polarised forward signaling drives stronger cell segregation than polarised reverse signaling.
• Non-polarised EphB2 activation leads to little if any cell segregation.
• Bidirectional forward and reverse signaling drives strong segregation by preventing intermingling in both directions.

## 1 Introduction

The formation of organised tissues requires that adjacent cell populations with a distinct tissue or regional identity form sharp borders, across which cells do not intermingle despite the intercalation of cells that occurs during growth and morphogenesis. Insights into underlying mechanisms have come from cell culture experiments in which different cell types are mixed and segregate from each other [1]. Initial studies identified mechanisms in which differences in homotypic affinity of distinct cell types drive cell segregation. For example, segregation can be driven by differential adhesion mediated by cadherin family members [[2], [3], [4]] or by differential cell surface contractility [[5], [6], [7]].

Subsequent work revealed another class of mechanisms in which cell responses to heterotypic interactions drive cell segregation and border sharpening [[8], [9], [10], [11]]. A mechanistic understanding has come from studies revealing a major role of Eph receptor and ephrin signaling in maintenance of tissue organisation in vertebrates [12,13]. Eph receptor tyrosine kinases and ephrins are subdivided into two classes based on sequence similarities and binding affinities: with some exceptions, EphAs bind to GPI-anchored ephrinA proteins, and EphBs to transmembrane ephrinB proteins [14]. Upon cell contact and binding of Eph to ephrin, both components cluster and transduce signals [15,16]. Eph receptors mediate ‘forward’ signaling which involves tyrosine kinase dependent pathways, and ephrins mediate ‘reverse’ signaling, which for ephrinB proteins involves their phosphorylation by cytoplasmic tyrosine kinases [15,16].

Since Ephs and ephrins that have high affinity are commonly expressed in complementary regions [14], signaling occurs at the heterotypic interface. This signaling can potentially act through multiple mechanisms to drive cell segregation and border sharpening [12,13,17]: by decreasing intercellular adhesion [18,19]; by increasing cortical tension [[20], [21], [22]]; or by cell repulsion, otherwise known as the contact inhibition of locomotion [[23], [24], [25], [26]]. The relative importance of these mechanisms likely depends upon tissue context, with differential tension or adhesion occurring where cells have sustained adhesive contact, and repulsion prominent for migratory cells. Forward and reverse signaling have each been shown to regulate cell adhesion and the actin cytoskeleton [16], and regulate many of the same targets of tyrosine phosphorylation [27]. This raises the question of whether unidirectional forward or reverse signaling, or bidirectional signaling, drives cell segregation. Initial work suggested that bidirectional signaling is required [10], but used truncated ephrins that may alter forward as well as reverse signaling [27]. Several recent studies have suggested that forward signaling is sufficient for cell segregation.

Studies of the ectoderm-mesoderm border in *Xenopus* have found that each tissue expresses both Ephs and ephrins, with complementary expression of high affinity pairs such that strongest activation occurs at the heterotypic interface [28]. Consequently, both forward and reverse signaling is bidirectional. Bidirectional forward signaling is sufficient to prevent intermingling of ectoderm and mesoderm [28] and acts by increasing heterotypic interfacial tension [21]. Thus forward signaling has a dominant role at a border at which forward signaling occurs on both sides. However, there could be a different situation at other borders, where there is forward signaling on one side and reverse signaling on the other [11,[29], [30], [31]].

Further insights have come from knockdown experiments [29,30] and mutant mice [32,33] in which there is mosaic expression of specific Ephs or ephrins. In the latter studies, mosaic knockout of ephrinB1 leads to segregation of expressing and non-expressing cells within the neural epithelium. Cell segregation also occurs in ephrinB1 mutants in which reverse signaling is disrupted, suggesting that forward signaling is sufficient [22]. In this mosaic situation, Eph activation is polarised in cells in contact with ephrinB1-expressing and non-expressing cells, whereas reverse signaling is not polarised since all cells are expressing Eph receptors. The lack of a contribution of reverse signaling may reflect a requirement for polarised activation, for example to elicit directional cell migration that drives segregation [24,26]. Nevertheless, non-polarised Eph or ephrin signaling could drive segregation by creating a difference in homotypic affinity, but this has not been directly tested.

We developed cell culture assays to analyse Eph-ephrin mediated cell segregation and border sharpening using HEK293 cell lines with stable overexpression of EphB2 or ephrinB1, termed EphB2 and ephrinB1 cells [24,27]. The results of experimental manipulations and measurements of cell behaviour revealed that heterotypic repulsion is the main driver of cell segregation of these migratory cells [26]. Since EphB2 and ephrinB1 cells each have a heterotypic repulsion response [26], they can be used to analyse whether forward and reverse signaling can both contribute to segregation. A recent study which used these cell lines proposed that forward signaling is sufficient to drive segregation [22], but did not test a role of reverse signaling.

Here, we test the role of reverse signaling, and of polarised versus non-polarised Eph and ephrin activation, in cell segregation and border sharpening. We find that polarised EphB2 activation drives stronger segregation than polarised ephrinB1 activation. However, bidirectional activation is required since cell segregation is greatly decreased when reverse signaling is disrupted. Non-polarised EphB2 activation drives little if any segregation. These findings support the idea that bidirectional Eph-ephrin signaling enables cell segregation and can be mediated by forward and reverse signaling.

## 2 Results

### 2.1 ephrinB1-6F mutant activates EphB2 phosphorylation

**Fig. 1:**
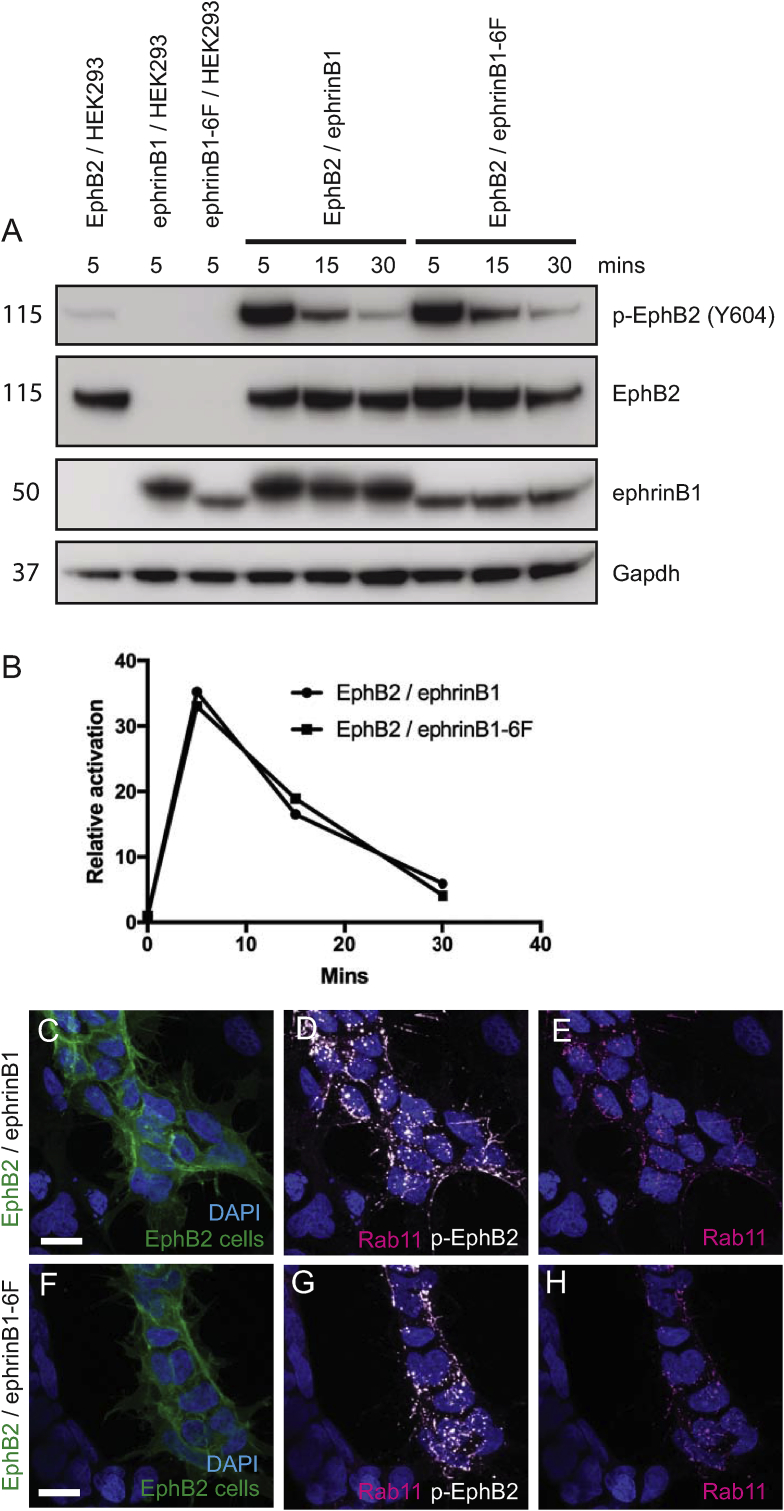
Activation of EphB2 by ephrinB1 and ephrinB1-6F. (A): Cells expressing EphB2, ephrinB1 or ephrinB1-6F, or control HEK293 cells, were mixed in different combinations in a tube and centrifuged to force them into contact. The cells were lysed at the indicated time points, then subjected to Western blot analysis to detect EphB2, phospho-EphB2 and ephrinB1, and Gapdh as loading control. (B): Quantitation of phospho-EphB2 detected, normalised to the amount of EphB2 protein. The low amount of endogenous ephrinBs in HEK293 cells elicit low level EphB2 phosphorylation. There is a similar time course of EphB2 phosphorylation after interaction with ephrinB1 or ephrinB1-6F cells, with a >30-fold increase by 5 min that progressively declines at 15 min and 30 min. (C-H): immunostaining of co-cultures of EphB2/ephrinB1 (C–E) and EphB2/ephrinB1-6F cells (F-H) to detect p-EphB2 and a marker of intracellular vesicles, Rab11. EphB2 cells co-express GFP (green signal in C, F). Activation of EphB2 with ephrinB1 or ephrinB1-6F leads to translocation from the cell surface to intracellular vesicles. Scale bar, 10 μm. (For interpretation of the references to colour in this figure legend, the reader is referred to the Web version of this article.)

**Fig. S1:**
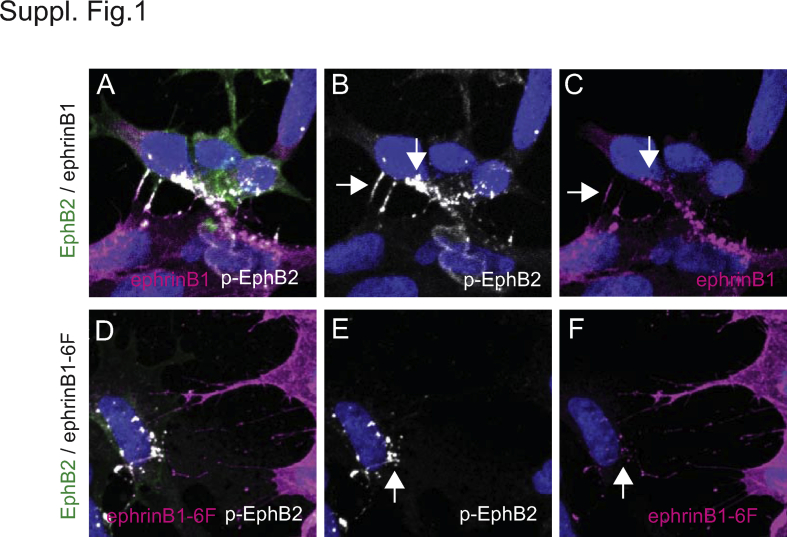
Relationship between activated EphB2 and wild type or mutant ephrinB1. (A-F): Immunostaining of cell cultures to detect ephrinB1 and p-EphB2. In (A and D), the green signal is GFP which marks EphB2 cells. Both in EphB2/ephrinB1 (A-C) and EphB2/ephrinB1-6F (D-F) cultures, p-EphB2 and ephrinB1 are detected in retraction fibres and in vesicles (arrows). There is overlap between signal for p-EphB2 and ephrinB1, reflecting that after binding they are endocytosed as an intact complex.

Since studies with truncated ephrinB1 suggest that the cytoplasmic domain is required for normal activation of forward signaling [27], it is important to ascertain whether ephrin mutants designed to disrupt reverse signaling still activate EphB2. Reverse signaling of ephrinB proteins is activated by clustering upon binding to Eph receptors, which leads to phosphorylation of conserved tyrosine residues by src family kinases [34,35]. In addition, ephrinB proteins can regulate cell behaviour via intracellular pathways downstream of PDZ domain proteins that bind to the C-terminal motif, but their binding is independent of, or even suppressed by interaction with Eph receptor [[36], [37], [38]]. We therefore generated a HEK293 cell line expressing an ephrinB1 mutant which has all cytoplasmic tyrosine residues mutated (ephrinB1-6F), as this is predicted to not mediate a response to EphB-expressing cells, but still act as a ligand to activate forward signaling. To test whether this is the case, we carried out Western blot analysis to detect EphB2 tyrosine phosphorylation after mixing EphB2 cells with either HEK293 cells, ephrinB1 cells or ephrinB1-6F cells. To achieve synchronous interactions, the cells were mixed in a tube and briefly centrifuged to bring them rapidly into contact. In EphB2/HEK293 cell controls, a low level of EphB2 phosphorylation is detected (Fig. 1A), reflecting that there is low level expression of endogenous ephrinBs in HEK293 cells [26]. We found that when EphB2 and ephrinB1 cells are mixed, there is a >30-fold increase in EphB2 phosphorylation by 5 min of cell contact, that declines by 15 min and is close to basal levels by 30 min (Fig. 1A, B). We found the same level and time course of EphB2 tyrosine phosphorylation following interaction with ephrinB1-6F cells compared with ephrinB1 cells (Fig. 1A, B). The transient nature of EphB2 activation is likely due to the endocytosis of Eph-ephrin complexes [39,40] which by removing EphB2 from the cell surface renders it unavailable for further activation. Indeed, immunostaining of cells shows that upon activation most EphB2 is translocated from the cell surface to intracellular vesicles, and is also detected in retraction fibres that are or have recently been in contact with ephrinB1 cells; endocytosis occurs mainly in the forward direction in these cells (Fig. 1C–H). As anticipated from previous work, ephrinB1 and ephrinB1-6F co-localise with p-EphB2 in vesicles in EphB2 cells, and in some cases in retraction fibres (Suppl. Fig. 1A–F).

### 2.2 ephrinB1-6F mutant mediates forward but not reverse cell repulsion

**Fig. 2:**
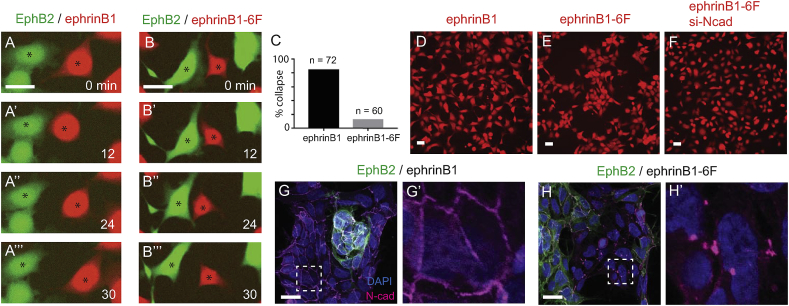
Unidirectional repulsion of EphB2 cells by ephrinB1-6F cells. (A, B): Time lapse movies of assays in which EphB2 cells (labelled green) were mixed with ephrinB1 cells or ephrinB1-6F cells (red) (Movies 1 and 2, respectively). Snapshots are shown of bidirectional repulsion of EphB2 and ephrinB1 cells (indicated with asterisk, A) and unidirectional repulsion of an EphB2 cell by ephrinB1-6F cell (B). (C): Quantitation of % collapse response of ephrinB1 cells compared with ephrinB1-6F cells after interaction and repulsion of EphB2 cells. (D–F): Homotypic cultures of ephrinB1 or ephrinB1-6F cells. Due to homotypic repulsion, ephrinB1 cells remain dispersed in low density culture (D). In contrast, ephrinB1-6F cells form aggregates since homotypic repulsion is diminished (E). This aggregation is diminished following knockdown of N-cadherin (F). (G, H): Immunostaining for N-cadherin (purple signal) after mixing EphB2 cells (green) with ephrinB1 cells (G) or ephrinB1-6F cells (H); higher magnification of the indicated area is shown in G’, H’. N-cad is detected along the homotypic interface of ephrinB1 cells, but is clustered at the interface of ephrinB1-6F cells. Scale bars, 20 μm. (For interpretation of the references to colour in this figure legend, the reader is referred to the Web version of this article.)

Although ephrinB1-6F elicits the same level of EphB2 tyrosine phosphorylation as ephrinB1, it remained possible that there is a change in some other aspect of activation that alters the cellular response. We therefore carried out time lapse studies in low density culture and analysed cell behaviour. As shown previously [26], interaction of EphB2 and ephrinB1 cells triggers repulsion in both the forward and reverse direction, manifested by a rapid cytoskeletal collapse at the site of contact to transiently form a flat or concave cell surface, accompanied by directional movement of the cell (Fig. 2A; Movie 1). Quantitation revealed that a collapse response occurs in ephrinB1 cells in 85% of heterotypic collisions in which EphB2 cells have a collapse response (Fig. 2C). We found that ephrinB1-6F cells also trigger a collapse response of EphB2 cells (Fig. 2B; Movie 2), but in only 13% of these interactions do the ephrinB1-6F cells themselves have a collapse response (Fig. 2C). In addition, we find a change in the homotypic repulsion of ephrinB1-expressing cells which is likely due to low level endogenous expression of EphB receptors that can activate reverse signaling [26]. Homotypic repulsion between ephrinB1-expressing cells is greatly diminished in ephrinB1-6F cells, and they form aggregates in low density culture, whereas such aggregation does not occur for ephrinB1 cells (Fig. 2D, E).

Supplementary video related to this article can be found at https://doi.org/10.1016/j.yexcr.2019.04.040.

**Fig. S2:**
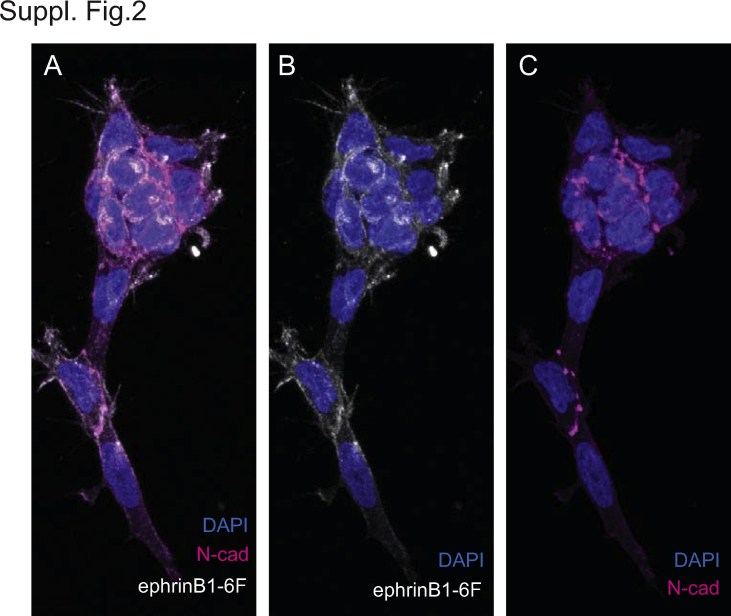
Relationship between ephrinB1-6F and N-cadherin. (A-C): immunostaining of a cluster of ephrinB1-6F cells to detect N-cadherin and ephrinB1-6F. N-cadherin is present in puncta at cell-cell contacts whereas ephrinB1-6F is more widely distributed and does not specifically co-localise with N-cadherin. These data are consistent with regulation of N-cadherin localisation by ephrinB1 signaling rather than formation of a complex between these proteins.

The following is the supplementary data related to this article:

To further characterise the changes in behaviour of ephrinB1-6F cells, we carried out immunostaining for cadherin, since previous work has shown that Eph signaling can act through actomyosin contraction to decrease adhesion by decreasing the clustering of cadherin at cell contact sites [18]. For HEK293 cells, N-cadherin is the predominant classical cadherin expressed, and was shown to antagonise Eph- and ephrin-mediated repulsion [26]. As found previously [26], N-cadherin is uniformly distributed along the homotypic and heterotypic interfaces of cells in EphB2/ephrinB1 cultures (Fig. 2G, G’). We found a striking change in ephrinB1-6F cells, in which N-cadherin forms clusters at the homotypic contacts between cells (Fig. 2H, H’). Immunostaining reveals no specific correlation between N-cadherin puncta and ephrinB1-6F location (Suppl. Fig. 2A–C), suggesting that this involves altered signaling rather than co-clustering. To test whether N-cadherin mediates adhesion of ephrinB1-6F cells, we carried out knockdown of N-cadherin and found that the aggregation in homotypic culture is decreased (Fig. 2F). Thus in the absence of phosphotyrosine-dependent reverse signaling there is a decrease in homotypic repulsion and increase in N-cadherin clustering that mediates cell adhesion. Clustering of N-cadherin may therefore be antagonised by phosphotyrosine-dependent reverse signaling, and/or promoted by PDZ domain-dependent signaling that is intact in the ephrinB1-6F mutant.

Taken together, these findings reveal that ephrinB1-6F cells have a greatly decreased homotypic and heterotypic cell repulsion response, but still elicit EphB2 activation and a strong repulsion response in EphB2 cells. We can therefore use ephrinB1-6F cells to address the question of whether repulsion mediated by reverse signaling can contribute to cell segregation and border sharpening.

### 2.3 Reverse signaling contributes to cell segregation and border sharpening

**Fig. 3:**
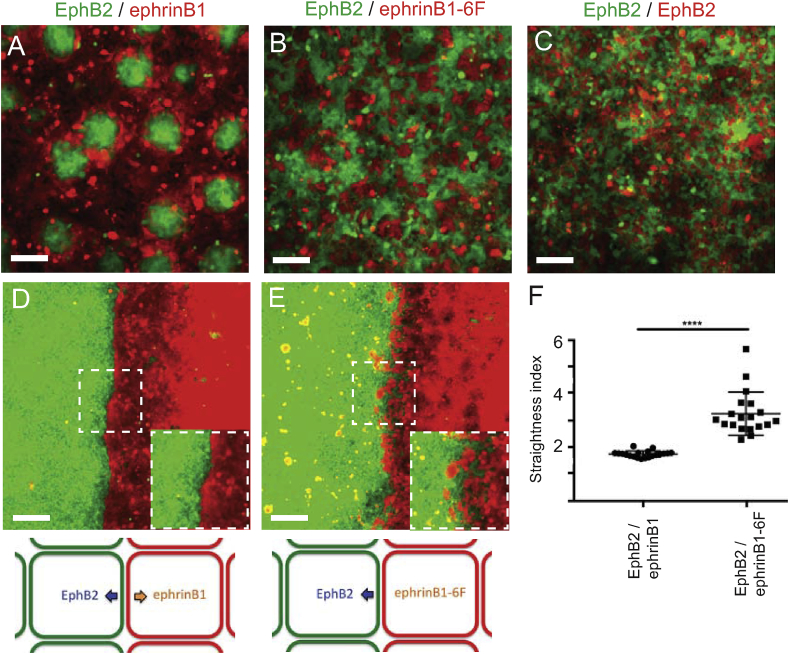
Decreased segregation and border sharpening by ephrinB1-6F cells. (A-C): Segregation assays were carried out with EphB2 cells (green) mixed with ephrinB1 cells (red) (A), ephrinB1-6F cells (B) or EphB2 cells (negative control, C). In EphB2 / ephrinB1 cell assays, EphB2 cells segregate to form compact clusters. Less extensive segregation occurs in EphB2 / ephrinB1-6F cell assays. (D, E): Boundary assays were carried out for EphB2 / ephrinB1 cells (D) and EphB2/ephrinB1-6F cells (E). A higher magnification view of the indicated area is in the bottom right of the panel. (F): Border sharpness was quantitated by comparing the length of the border with that of a straight line. The border is less sharp for EphB2 / ephrinB1-6F cells than for EphB2 / ephrinB1 cells. ****, p<0.0001 . Scale bars, 100 mm.

We tested the role of reverse signaling in cell segregation assays in which cells are mixed and plated at non-confluent density [24,26]. In assays with EphB2 and ephrinB1 cells, there is extensive segregation leading to formation of clusters of EphB2 cells with sharp borders surrounded by ephrinB1 cells (Fig. 3A). When EphB2 and ephrinB1-6F cells are mixed, there is segregation (compare with negative control, Fig. 3C) but significantly less than for EphB2 and ephrinB1 cells (Fig. 3B). Notably, the interface between EphB2 and ephrinB1-6F cell clusters is not sharp and there is no longer an inside-out organisation of segregation in which clusters of EphB2 cells are surrounded by ephrinB1 cells.

To quantitate border sharpening, we plated cells on opposite sides of a barrier, which is then removed to allow the populations to migrate and meet to form a border [26]. In assays with EphB2 and ephrinB1 cells, the initially ragged border becomes sharp (Fig. 3D), whereas EphB2 and ephrinB1-6F cells do not form a sharp border (Fig. 3E). We quantitated border sharpness by measuring the length of the border and found that in assays with EphB2 and ephrinB1-6F cells, there is a significant decrease in border sharpening compared with EphB2 and ephrinB1 cells (Fig. 3F).

### 2.4 Loss of heterotypic repulsion of ephrinB1 cells disrupts segregation

**Fig. 4:**
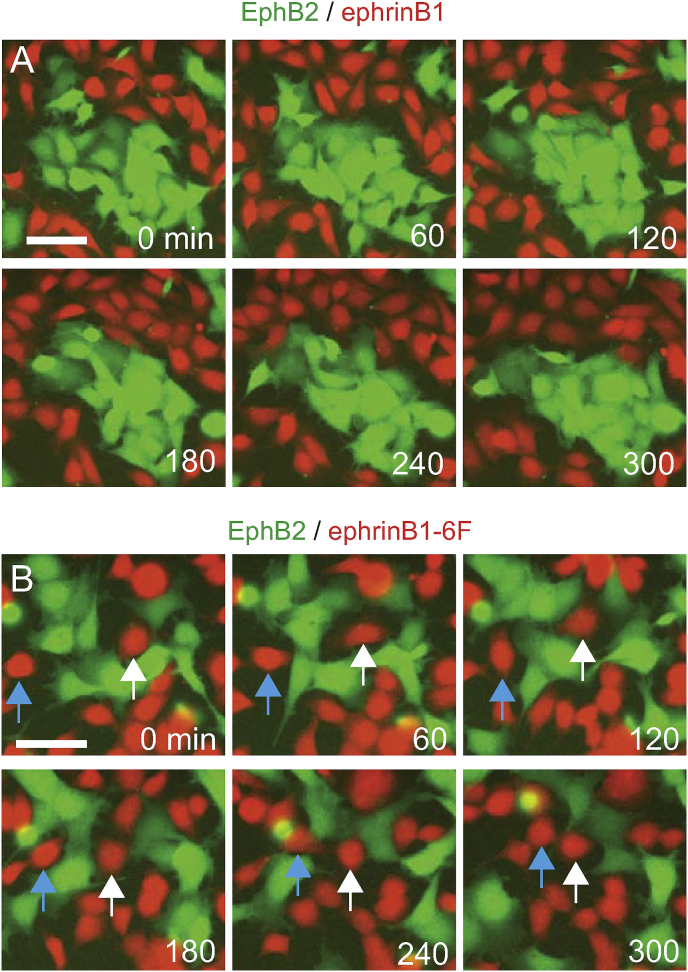
Movies of segregation assays. (A, B) Snapshots of time lapse movies at late stages of segregation of EphB2 and ephrinB1 cells (A) and EphB2 and ephrinB1-6F cells (B); Movies 3 and 4, respectively. In EphB2/ephrinB1 assays, the cluster of EphB2 cells is stable throughout the 300 min period analysed. In EphB2/ephrinB1-6F assays, small clusters of EphB2 cells form but are not stable. EphB2 cells have heterotypic repulsion responses, but an ephrinB1-6F cell (white arrow) fails to move away and segregate from EphB2 cells, and another ephrinB1-6F cell (blue arrow) intercalates between EphB2 cells. Scale bars, 50 μm. (For interpretation of the references to colour in this figure legend, the reader is referred to the Web version of this article.)

These findings raise the question of why segregation is greatly decreased when reverse signaling is absent, even though forward signaling and heterotypic repulsion of EphB2 cells still occurs. To address this, we analysed time lapse movies at late stages of segregation when cells are at high density. We found that in assays with EphB2 and ephrinB1 cells, the clusters of EphB2 cells are stable and there is no intermingling with ephrinB1 cells (Fig. 4A; Movie 3). In contrast, in assays with EphB2 and ephrinB1-6F cells, ephrinB1-6F cells do not actively migrate away from EphB2 cells upon contact (Fig. 4B, white arrow; Movie 4). Furthermore, ephrinB1-6F cells sometimes move into EphB2 cell clusters and break them up (Fig. 4B, blue arrow; Movie 4). Taken together, these findings suggest that decreased segregation occurs because ephrinB1-6F cells elicit repulsion of EphB2 cells but are not themselves repelled. In contrast, ephrinB1 cells have a heterotypic repulsion response and consequently do not mingle into forming EphB2 cell clusters.

Supplementary video related to this article can be found at https://doi.org/10.1016/j.yexcr.2019.04.040.

The following are the supplementary data related to this article:

### 2.5 Effect of decreased polarisation of forward and reverse signaling on segregation

**Fig. 5:**
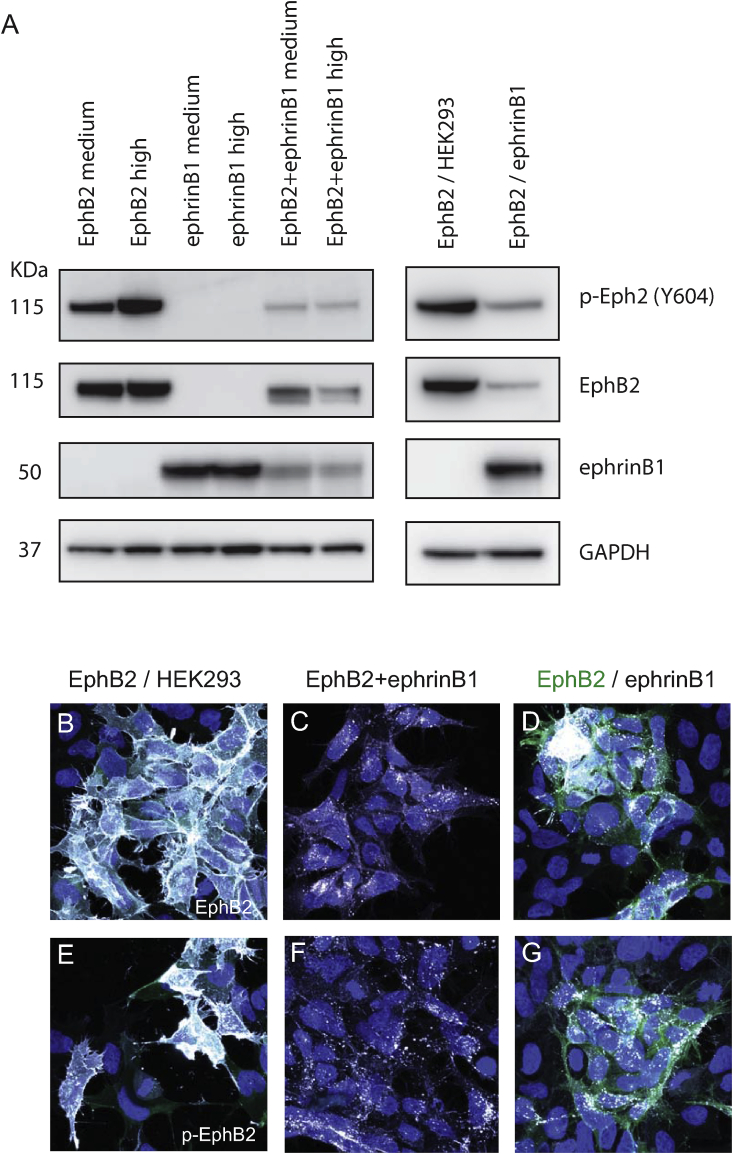
Analysis of EphB2 levels and activation in co-culture assays. (A): Cells were plated in different combinations at medium density (19,000 cells/cm^2^) or high density (38,000 cells/cm^2^), as indicated, and allowed to interact for 24 h to reach a steady state level of EphB2 activation as occurs in segregation assays. Left panel: EphB2 cells have basal activation by endogenous ephrinBs that is increased at higher cell density. In EphB2+ephrinB1 cells, the steady state level of EphB2 and phospho-EphB2 decreases, likely due to repeated homotypic contacts that lead to endocytosis and degradation. Right panel: There is a decrease in steady state levels of EphB2 and phospho-EphB2 after co-culture of EphB2 cells and ephrinB1 cells compared with EphB2 cells alone. (B–G): Cells were immunostained for EphB2 (B–D) or phospho-EphB2 (E–G). In EphB2 cells co-cultured with HEK293 cells, most EphB2 and phospho-EphB2 is on the cell surface (B, E), whereas it is mainly in intracellular vesicles in EphB2+ephrinB1 cells (C, F). EphB2 and phospho-EphB2 is detected mainly in intracellular vesicles in EphB2 cells co-cultured with ephrinB1 cells (D, G).

Mosaic loss of ephrin function leads to polarised activation of Eph receptors in cells that contact ephrin-expressing and non-expressing cells, whereas ephrin activation is not polarised since Eph receptors are uniformly expressed. Conversely, mosaic loss of Eph receptor function leads to polarised activation of ephrins and non-polarised activation of Eph receptor. Interpretation of the role of forward and reverse signaling in mosaic experiments thus depends upon whether non-polarised as well as polarised activation can drive strong cell segregation. To test this, we established a stable cell line in which EphB2 is overexpressed in the previously-generated ephrinB1 cell line (EphB2+ephrinB1 cells). We carried out Western blot analyses to determine the level of EphB2 and ephrinB1 expression when these cells are harvested for analysis at densities used in segregation assays, in which there are frequent homotypic collisions. We found that there is a lower level of EphB2 and ephrinB1 protein, and of phospho-EphB2, in co-expressing cells compared with homotypic cultures of EphB2 or ephrinB1-expressing cells (Fig. 5A, left panel). A potential explanation is that homotypic contacts between EphB2+ephrinB1 cells lead to activation, endocytosis and degradation that reduces the steady state level of Eph and ephrin protein. Indeed, we found that most EphB2 and phospho-EphB2 protein is present in vesicles within the cells (Fig. 5C, F), whereas it is mainly on the cell surface in EphB2 cells mixed with HEK293 cells (Fig. 5B, E). Furthermore, mixing of EphB2 and ephrinB1 cells followed by culture for 24 h at the density used for segregation assays also leads to endocytosis of EphB2 protein (Fig. 5D, G) and a major decrease in the level of EphB2 protein and EphB2 phosphorylation (Fig. 5A, right panel). Thus, the repeated heterotypic contacts that occur during cell segregation decrease the steady state level of EphB2 and ephrinB1 protein, that nevertheless is sufficient to trigger cell repulsion [26].

**Fig. 6:**
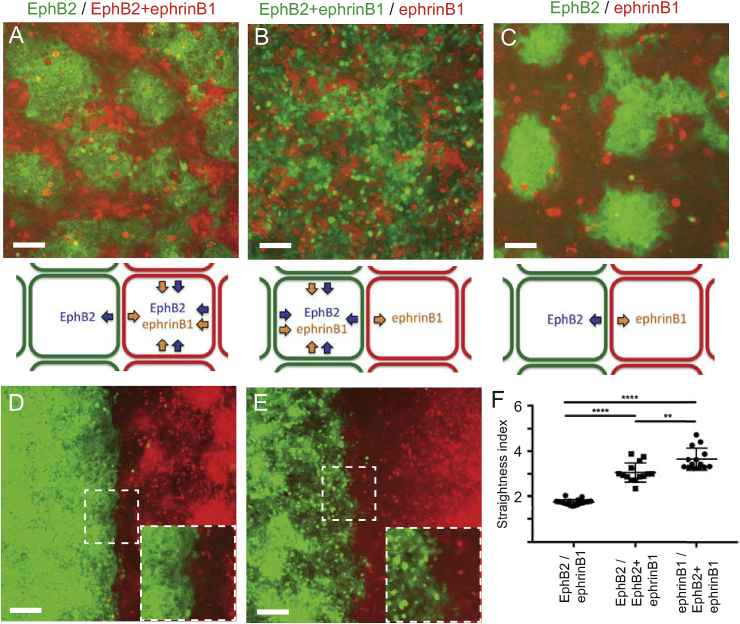
Effect of suppressing polarised Eph or ephrin activation on cell segregation and border sharpening. (A–C): Segregation assays with EphB2, ephrinB1 or EphB2+ephrinB1 cells mixed in different combinations. Segregation occurs in EphB2/EphB2+ephrinB1 cell assays in which activation of EphB2, but not of ephrinB1, is polarised (A). Segregation is less extensive in ephrinB1/EphB2+ephrinB1 cell assays in which activation of ephrinB1, but not of EphB2, is polarised (B). In control EphB2/ephrinB1 cell assays, EphB2 cells segregate to form compact clusters (C). Scale bars, 100 μm. (D, E): Boundary assays with EphB2/EphB2+ephrinB1 cells (D) and ephrinB1/EphB2+ephrinB1 cells. A higher magnification view of the indicated area is in the bottom right of the panel. (F): Quantitation of boundary assays. Border sharpness is decreased for EphB2/EphB2+ephrinB1 cells compared with EphB2/ephrinB1 cells, and further decreased for ephrinB1/EphB2+ephrinB1 cells. ****, p < 0.0001; **, p < 0.01. Scale bars, 100 μm.

We carried out segregation assays in which EphB2 cells are mixed with EphB2+ephrinB1 cells. In this situation, polarised forward signaling occurs in EphB2 cells activated by ephrinB1, whereas reverse signaling becomes less polarised since all cells are expressing EphB2. We found that there is strong segregation of the two cell populations (Fig. 6A). Interestingly, the EphB2 cell clusters do not become compacted, whereas they do in EphB2/ephrinB1 assays (Fig. 6C). This finding is consistent with evidence that compaction is due to asymmetry in the amount of cell repulsion since there is a more sustained directional migration following EphB2 activation compared with ephrinB1 activation [26]. We tested whether polarised forward signaling can drive border sharpening by carrying out boundary assays. We found that border sharpening occurs for assays with EphB2 cells confronted with EphB2+ephrinB1 cells but is less extensive than for EphB2/ephrinB1 cells (Fig. 6D, F). This decrease in border sharpening may reflect that ephrinB1 activation is non-polarised and/or that the steady state level of ephrinB1 is lower in the co-expressing cells (Fig. 5A).

In the reciprocal experiment, we mixed ephrinB1 cells with EphB2+ephrinB1 cells, and found that this leads to less extensive segregation (Fig. 6B) than for EphB2/EphB2+ephrinB1 (Fig. 6A). Likewise, in boundary assays there was a lower amount of border sharpening for ephrinB1/EphB2+ephrinB1 cells compared with EphB2/EphB2+ephrinB1 cells (Fig. 6E, F). In summary, segregation and border sharpening occurs in assays in which EphB2 activation is highly polarised, and less strongly in assays in which ephrinB1 activation is highly polarised. Since in the latter situation, EphB2 is activated by all cells, this argues that non-polarised forward signaling is not able to drive efficient segregation. These findings suggest that cell segregation is driven predominantly by polarised activation of EphB2.

## 3 Discussion

It has long been known that interactions between Eph receptors and ephrins lead to forward and reverse signaling [34,35], but remained unclear whether bidirectional signaling is required for cell segregation and border sharpening. Recent work has suggested that unidirectional Eph activation leading to polarised forward signaling is sufficient to drive cell segregation [22], but this was based on the results of mosaic knockout of ephrinB1 in which reverse signaling is non-polarised. However, interacting Ephs and ephrins are commonly expressed in complementary domains [14] which will lead to polarised forward and reverse signaling. Here, we have analysed the role of forward and reverse signaling in cell culture assays in which contact repulsion drives cell segregation and border sharpening. We find that polarised forward signaling alone is sufficient to drive cell segregation, but polarised forward and reverse signaling leads to greater segregation and border sharpening.

To analyse roles of reverse signaling, we created a cell line expressing ephrinB1-6F mutant, which we find has a greatly reduced heterotypic repulsion response, but still activates forward signaling and heterotypic repulsion of EphB2-expressing cells. There is a significant decrease in cell segregation and border sharpening in assays with EphB2/ephrinB1-6F cells compared with EphB2/ephrinB1 cells. Analysis of time lapse movies reveals that the decreased heterotypic repulsion of ephrinB1-6F cells leads to a failure to migrate away from EphB2 cells, and increased mixing into EphB2 cell territory. Likewise, previous work revealed a requirement for forward signaling since cells expressing truncated EphB2 mingle into ephrin-expressing territory [10]. A role of bidirectional signaling is supported by studies of the ectoderm-mesoderm boundary showing that manipulation of Eph and ephrin expression to create bidirectional forward signaling led to greater border sharpening than unidirectional forward signaling [21]. Our findings suggest that forward and reverse signaling can underlie bidirectional repulsion, in which reverse signaling prevents mingling of ephrinB1 cells into the forming clusters of EphB2 cells.

Since a number of studies have shown that polarised forward signaling is sufficient to drive segregation [22,28], this raises the question of the relative contribution of forward and reverse signaling when there is complementary expression of interacting Eph receptor and ephrin. We obtained insights from segregation assays in which cells co-expressing EphB2 and ephrinB1 are mixed either with EphB2 or ephrinB1 cells. In assays with EphB2+ephrinB1/ephrinB1 cells, the polarised activation of EphB2 is diminished due to co-expression of ephrinB1, but polarised activation of ephrinB1 still occurs. We find that in this situation there is greatly reduced segregation and border sharpening compared with EphB2/ephrinB1 cell controls. In contrast, strong segregation occurs in assays with EphB2+ephrinB1/EphB2 cells in which polarised activation of ephrinB1 is diminished, and polarised activation of EphB2 still occurs. Furthermore, there is greater border sharpening in this situation compared with experiments in which the polarisation of EphB2 activation is decreased. Although border sharpness is decreased for EphB2+ephrinB1/EphB2 compared with EphB2/ephrinB1, this may be due to a lower steady state level of ephrinB1 in the co-expressing cells in which activation and endocytosis occurs following both homotypic and heterotypic interactions. Alternatively, or in addition, the decreased polarisation of ephrinB1 activation at the heterotypic interface may decrease sharpening. These findings support the idea that polarised forward signaling is the principal driver of cell segregation. We propose that although polarised reverse signaling is less efficient in driving segregation, it contributes by diminishing the mingling of ephrin-expressing cells into territory expressing Eph receptor.

A corollary of this model is that there is a difference in the cellular response to activation of EphB2 compared with ephrinB1. Indeed, quantitative analyses revealed that EphB2 cells have more persistent directional migration than ephrinB1 cells during heterotypic repulsion [26]. The observation that over-expression of ephrinB1 in EphB2 cells disrupts segregation from ephrinB1 cells is consistent with the idea that polarised activation of EphB2 is more efficient than non-polarised activation in driving cell segregation. In support of this, computer simulations based on measurements of cell behaviour have shown that increased repulsion or tension at the heterotypic interface drives segregation much more efficiently than differences in homotypic cell affinity [21,26]. This requires a sufficient quantitative difference between heterotypic and homotypic repulsion, which is diminished when homotypic repulsion is increased by co-expression of Eph and ephrin (this study) or by cadherin knockdown [26].

Our findings raise the question of whether bidirectional signaling also contributes to cell segregation *in vivo*. A requirement for bidirectional signaling may depend upon the extent to which cell intermingling challenges the maintenance of segregated populations. In the cell culture assays used here, cells are migratory and bidirectional signaling is required to prevent Eph and ephrin-expressing cells from mingling into the other population. Likewise, forward signaling in migrating mesoderm cells is required to prevent them from invading underlying ectoderm tissue [25,28]. Although active cell migration does not occur within epithelial tissues, there is mixing of cells that challenges boundaries, for example at the borders of hindbrain segments [41,42] where it is restricted by Eph-ephrin signaling [11,[29], [30], [31]]. This intermingling occurs through cell proliferation which can cause cells to intercalate across the border. In some tissues, a further challenge comes from morphogenetic movements such as convergent-extension which drive extensive cell intercalation during the period when sharp borders are forming in the neural epithelium [43]. It will therefore be important to uncover whether bidirectional forward and reverse signaling is required to form and maintain sharp borders in epithelial tissues. This will require the generation of mutations that selectively disrupt forward and reverse signaling in an Eph-ephrin pair that mediate border sharpening.

## 4 Materials and methods

### 4.1 Cell culture assays

Cells were cultured at 37 °C with 5% CO_2_ in DMEM supplemented with 10% fetal calf serum, glutamine and antibiotics. EphB2-expressing and ephrinB1-expressing HEK293 cell lines have been described previously [24]. A cell line co-expressing EphB2 and ephrinB1 was generated by transfection of the ephrinB1 cell line with EphB2 expression construct. A HEK293 cell line was generated that expresses the ephrinB1-6F mutant. Expression levels of ephrinB1-6F or EphB2 proteins in these cell lines were determined by both Western blotting and immunocytochemistry using anti-ephrinB1 or anti-EphB2 antibodies (R&D systems, AF473 or AF467, 1:2000 for Western blot, and 1:40 for immuno-staining). If cells are found to have heterogeneous expression, FACS and re-cloning are used to re-establish batches of cells with the same expression level.

To label cells for assays, they were treated with CMFDA (green) or CMRA (red) cell tracker dyes (Molecular Probes, Invitrogen) and then dissociated with Accutase (Sigma). In segregation assays [26], red and green labelled cells were mixed in equal proportions, plated on a fibronectin-coated coverglass chambered slide (Lab-Tek) at a density of 200,000 cell/cm^2^ and cultured for 48 h before fixation. To visualise individual cell responses, 20,000 cells were placed into each well (0.7 cm^2^) of an 8-well chambered slide, and imaged using a Nikon Ti2 microscope with Photometrics PrimeBSI sCMOS camera with a Plan Apochromat 20x/0.75NA objective. Images were taken every 3 min and were processed using ImageJ. In boundary assays [26], a two well culture insert (Ibidi) was placed onto a fibronectin-coated chambered slide and 88 μl of labelled cells put into each side at a concentration of 1 million cells/ml. Cells were incubated at 37 °C for 4 h before lifting the barrier and addition of fresh medium, and then incubated for a further 40 h to allow the cell populations to meet and interact at the boundary. Images were taken by the Nikon Ti2 microscope with a Plan Fluor 10x/0.3NA objective.

### 4.2 Quantitation of cell behaviour

Border sharpness was quantitated in boundary assays by measuring the length of a series of segments of the boundary from thresholded grayscale images of the EphB2 cell population [26]. This value was converted to a sharpness index by dividing it by the minimum border length attained by a straight line [44]. We analysed low density cell assays to visualise individual cell behaviour. Cells were tracked and heterotypic collisions scored for whether there is a collapse response of one or both cells.

### 4.3 Activation of EphB2 by cell mixing

Different cell lines were harvested separately by trypsinisation and the resulting cell suspensions adjusted to 6 × 10^6^ cells/ml. 0.25 ml each of two cell lines were then mixed in different combinations in a 1.5 ml eppendorf tube so that there was a total of 3 × 10^6^ cells. These cell mixtures were then centrifuged at 180 rpm for 45 s to force the cells into contact within a pellet. The pellets were then incubated at 37 °C for the appropriate time. Following the incubation period, cells were lysed with RIPA buffer (50 mM Tris/HCl pH 8, 150 mM NaCl, 1% Triton-X100, 0.5% Sodium Deoxycholate, 0.1% SDS) supplemented with protease inhibitors (Sigma Aldrich) on ice. The lysates were briefly sonicated and then cleared by centrifugation at 13,300 rpm for 15 min at 4 °C. The BCA assay was used to determine the protein concentration of the samples. Antibodies used for Western blot analysis were to detect: EphB2 and Gapdh from Thermo Fisher (371700, MA5-15738, respectively); phosphorylated EphB2-Y594 from Abcam (ab61791); ephrinB1/2/3 from Santa Cruz (sc-910).

### 4.4 Immunocytochemistry

Cells were fixed for 15 min in 4% paraformaldehyde at 37 °C, washed three times in PBS and stored at 4 °C prior to immunostaining. Cells were washed twice in PBT (0.1% Tween 20 in PBS), blocked for 30 min and then incubated for 2–3 h with primary antibodies in 2.5% goat serum, 1% DMSO in PBT. After washing eight times in PBT during 1 h, cells were incubated for 2 h in secondary antibody (donkey anti-mouse Cy5-conjugated and donkey anti-rabbit Cy3-conjugated, Jackson ImmunoResearch, 1:400) together with DAPI nuclear counterstain. The cells were then washed eight times in PBT and mounted in FluorSave. Immunostained cells were imaged using a Zeiss LSM700 confocal microscope and images processed using ImageJ. The antibodies used were to detect: Pan-cadherin (Sigma, C-1821, 1:200); EphB2 (see above, 1:300); phospho-EphB2 (see above, 1:300); Rab11 (BD Biosciences; 610656).

### 4.5 Gene knockdown

siRNAs were from Dharmacon (N-cadherin On-Target plus SMART pool and Non-targeting siRNA-SMART pool). A non-targeting siRNA was used as negative control. 60 pmol of siRNA was transfected with Lipofectamine RNAiMAX (Invitrogen) according to manufacturer's instructions. The transfected cells were incubated for 48 h before re-plating for assays.
